# Supplementary material for: β-amyloid monomer scavenging by an anticalin protein prevents neuronal hyperactivity in mouse models of Alzheimer’s Disease
Source: Nat Commun. 2024 Jul 10;15:5819. doi: 10.1038/s41467-024-50153-y (PMC11237084; doi:10.1038/s41467-024-50153-y)
Supplement: Supplementary file 1 — Supplementary Information [file 41467_2024_50153_MOESM1_ESM.pdf]

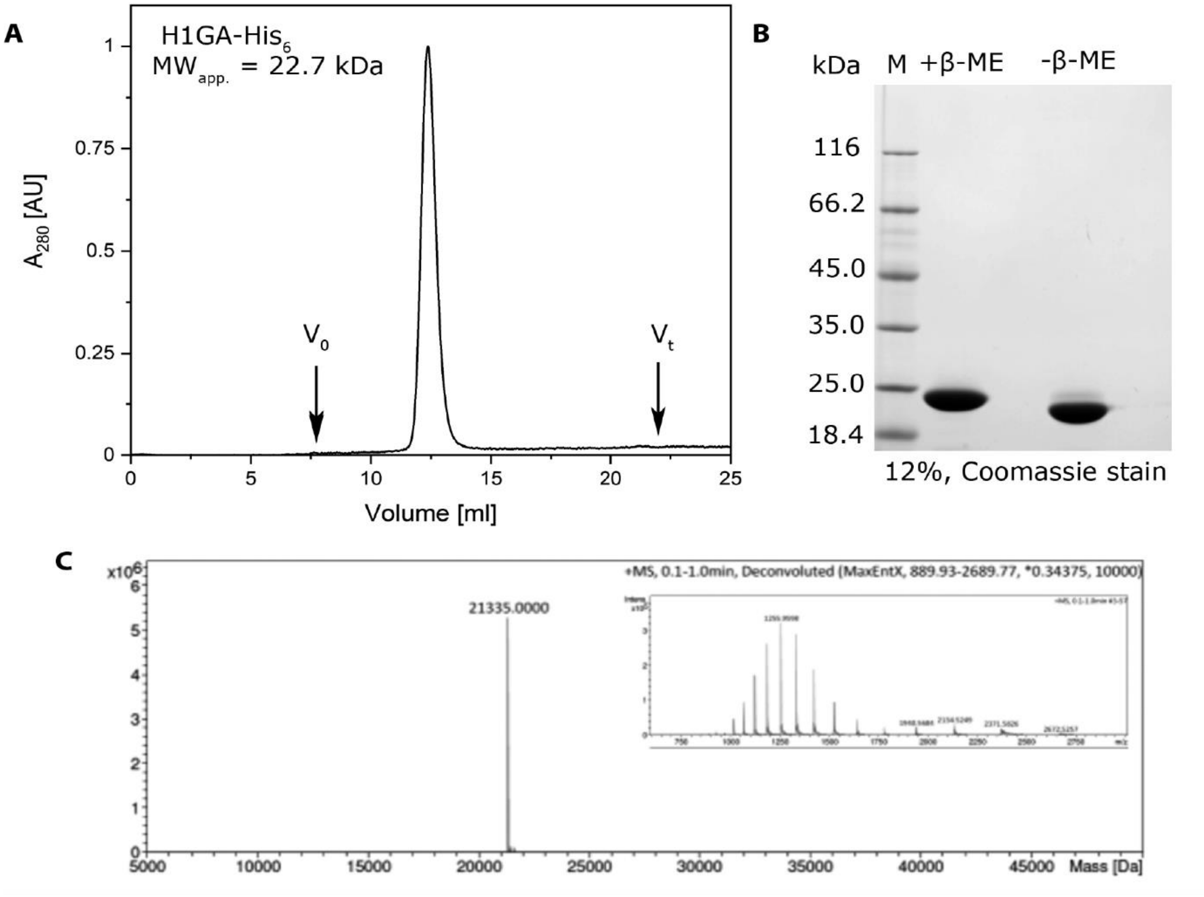
A
H1GA-His6
MWapp. = 22.7 kDa
A280 [AU]
V0
Vt
Volume [ml]
B
kDa
M
+β-ME
-β-ME
116
66.2
45.0
35.0
25.0
18.4
12%, Coomassie stain
C
x10^6
+MS, 0.1-1.0min, Deconvoluted (MaxEntX, 889.93-2689.77, *0.34375, 10000)
21335.0000
Mass [Da]

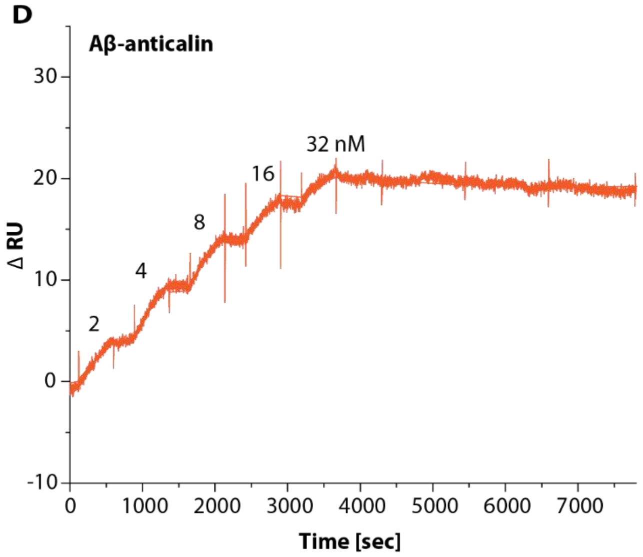
D
Aβ-anticalin
Δ RU
2
4
8
16
32 nM
Time [sec]

Fig. S1

Analysis of the Aβ-anticalin, related to Fig. 1: (**A**) Analytical SEC of the Aβ-anticalin (H1GA) confirming its monovalent and monodisperse state with an apparent molecular mass (~21.7 kDa). (**B**) SDS-PAGE analysis of the Aβ-anticalin under reducing (+β-ME) and non-reducing (-β-ME) conditions. The slightly enhanced electrophoretic mobility in the latter case is in agreement with the single structural disulfide bond of this engineered lipocalin protein. (**C**) ESI-mass spectrum of the purified recombinant Aβ-anticalin (calculated mass: 21335.18 Da, including the loss of two H-atoms due to the formation of one disulfide bond). (**D**) Real-time SPR analysis of the Aβ-anticalin probed against the covalently immobilized Aβ(1-40) peptide using single-cycle kinetics, revealing a K_D value of 0.54 nM ± 0.16.

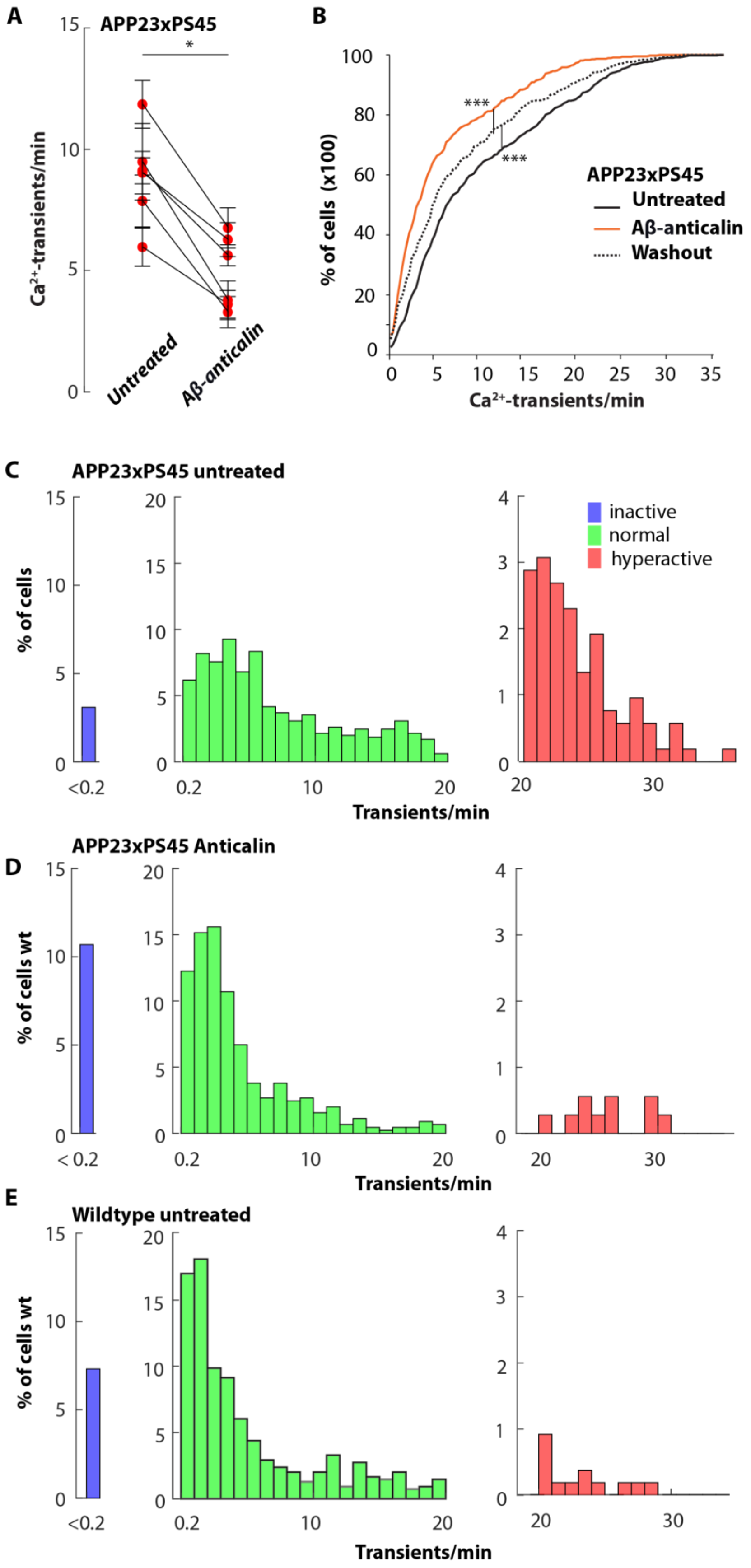

A
APP23xPS45
Ca2+-transients/min
Untreated
Aβ-anticalin
B
% of cells (x100)
Ca2+-transients/min
APP23xPS45
Untreated
Aβ-anticalin
Washout
C
APP23xPS45 untreated
% of cells
inactive
normal
hyperactive
Transients/min
APP23xPS45 Anticalin
D
% of cells wt
Transients/min
E
Wildtype untreated
% of cells wt
Transients/min

Fig. S2.

Suppression of neuronal hyperactivity by the Aβ-anticalin, related to Fig 1: (**A**) Neuronal activity in the hippocampal CA1 region of *APP23xPS45* mice before (*left*) and during (*right*) the targeted application of the Aβ-anticalin (10 µM). Each dot represents the average of one mouse under baseline conditions (left) and during the application of the Aβ-anticalin. Error bars depict SEM. (**B**) Cumulative distribution of neuronal activities in untreated (*black solid*) and treated (*orange*) *APP23xPS45* mice as well as after a washout of 5-10 minutes (*black dashed line*). (**C**) Histogram of the neuronal activity in the hippocampal CA1 region of untreated *APP23xPS45* mice. Hypoactive cells (<0.2 transients/min) are indicated in blue, normal neurons (0.2-20 transients/min) in green and hyperactive cells (>20 transients/min) in red (data from n = 648 cells in 6 mice). (**D**) Same as (**C**) during the application of 10 µM Aβ-anticalin (n=486 cells in 6 mice). (**E**) Same as (**C**) for untreated wild type mice (n = 449 cells in 7 mice). * $P< 0.05$, *** $P<0.0001$. Wilcoxon signed-rank test (A) or Kolmogorov-Smirnov-test (B).

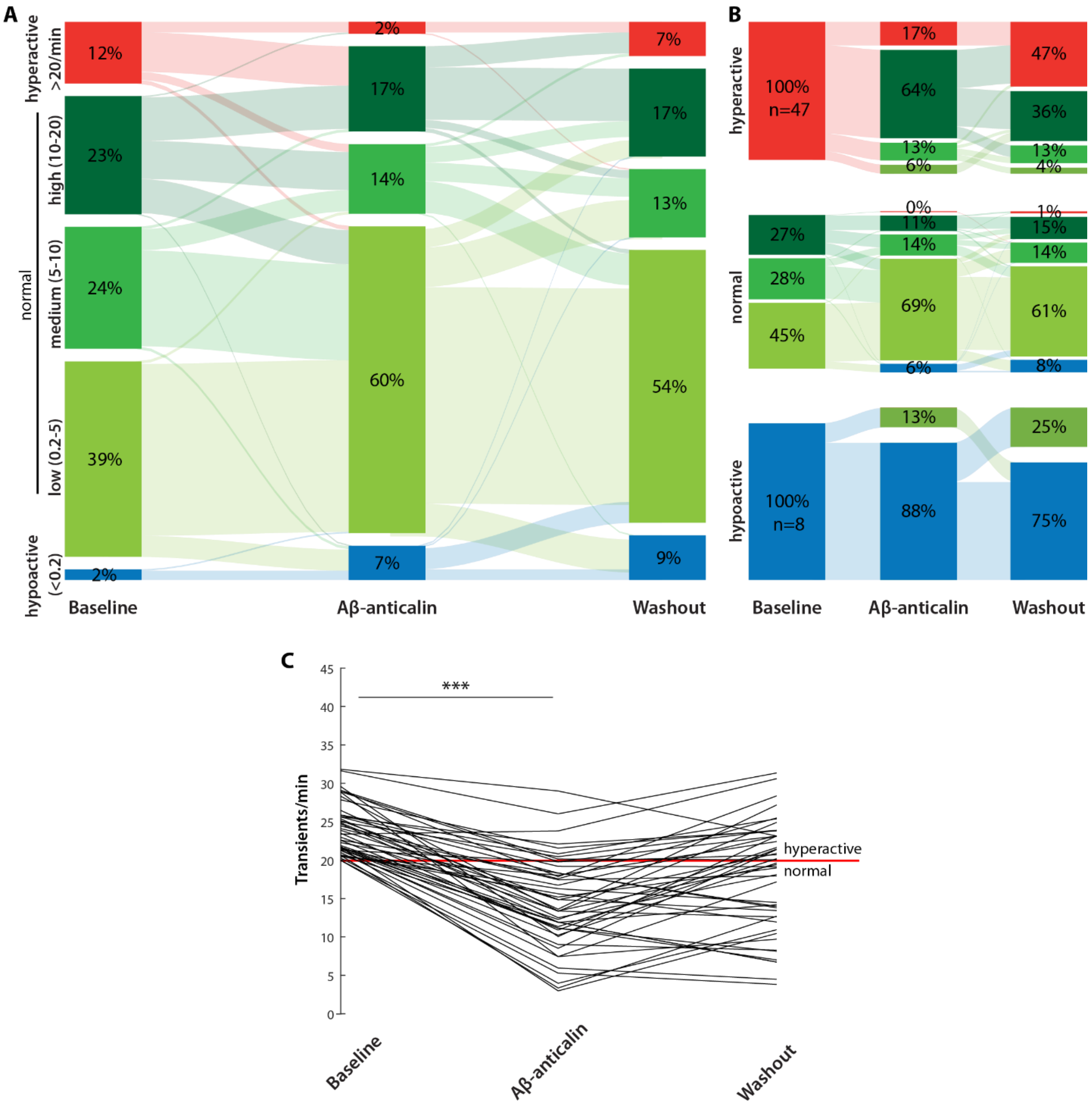

Fig. S3.

Neuronal activity reduction by the Aβ-anticalin, related to Fig 1: (**A**) Alluvial plot showing the number of Ca^{2+}-transients for all neurons in young *APP23xPS45* mice which could be followed during baseline (*left*), Aβ-anticalin application (*middle*) and washout (*right*). n=387 cells in 6 mice. Cells were classified, according to their baseline activity, as hyperactive (*red*), normal (*green*) or hypoactive (*blue*). (**B**) Alluvial plots for the three subgroups defined in (A). Hyperactive (*top*), normal (*middle*) and hypoactive cells (*bottom*). (**C**) Number of Ca^{2+}-transients for all neurons in *APP23xPS45* mice classified as hyperactive during baseline (l*eft*), Aβ-anticalin application (*middle*) and washout (*right*). Each line represents an individual neuron (n=47 in 6 mice). *** P<0.001.

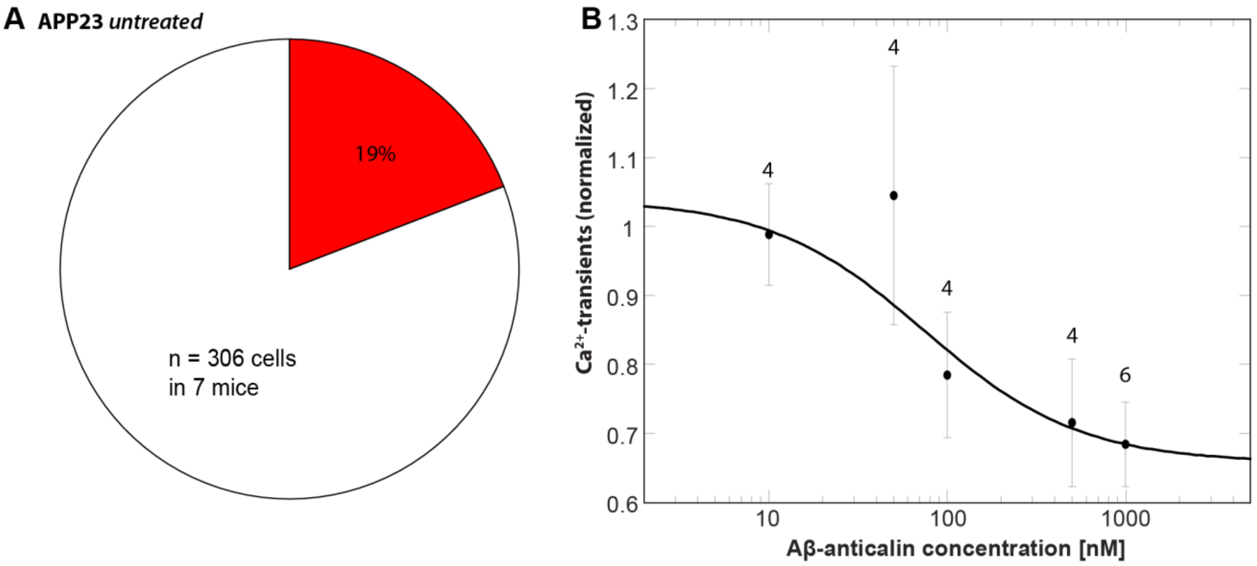

Fig S4.

Dose-response curve of Aβ-anticalin-dependent block of hyperactivity in *APP23* mice, related to Fig. 1. (A) Pie chart depicting the percentage of hyperactive neurons in non-plaque bearing *APP23* mice. (**B**) Neuronal activity during the application of the Aβ-anticalin, normalized to the respective baseline. Results for the application of 10nM (N=4 mice), 50 nM (N=4), 100nM (N=4), 500nM (N=4), and 1µM (N=6) in the application pipette. Data points correspond to mean values and error bars depict SEM. Curve fit was accomplished by non-linear regression, using SEM data weight, according to the Equation of Mass Law, yielding a half-maximal inhibitory concentration (corresponding to the apparent dissociation constant, K_D), IC_{50} = 75 nM.

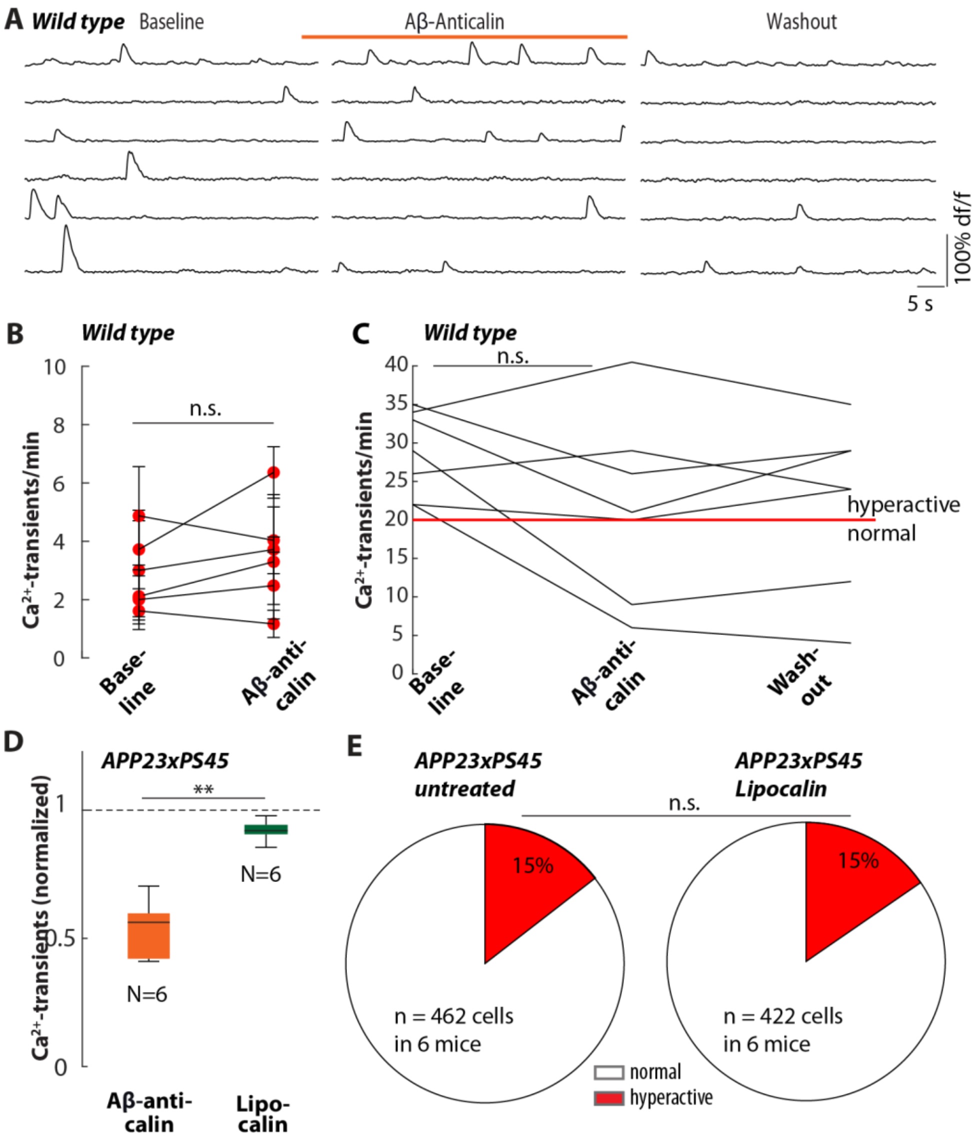

Fig. S5.

No effect of Aβ-anticalin in wild-type mice or of wild-type lipocalin in *APP23xPS45* mice, related to Fig 1: (**A**) Ca^{2+}-transients from six representative hippocampal CA1 pyramidal neurons of a wild-type mouse under baseline conditions, during the application of the Aβ-anticalin (10 µM) and after washout. (**B**) Summary data of the *in vivo* experiments in (A). Red dots represent the mean of one animal (N=6), error bars depict SEM. (**C**) Number of Ca^{2+}-transients for all neurons in the experiment in (A), which were classified as hyperactive during baseline (*left*), Aβ-anticalin application (*middle*), and washout (*right*). Each line represents an individual neuron (n=7 cells in 6 mice). (**D**) Comparison of the average number of Ca^{2+}-transients during the application of 10 µM Aβ-anticalin (*orange*, N=6 animals) or wild-type lipocalin (*green*, N=6), normalized to the respective baseline activity. (**E**) Pie charts depicting the percentage of hyperactive cells in untreated (*left*) and lipocalin-treated (*right*) *APP23xPS45* mice. Source data for Fig. S5D are provided as a Source Data file. * P<0.05, n.s. not significant, Wilcoxon signed-rank test (B, C), Wilcoxon rank sum test (D and E).

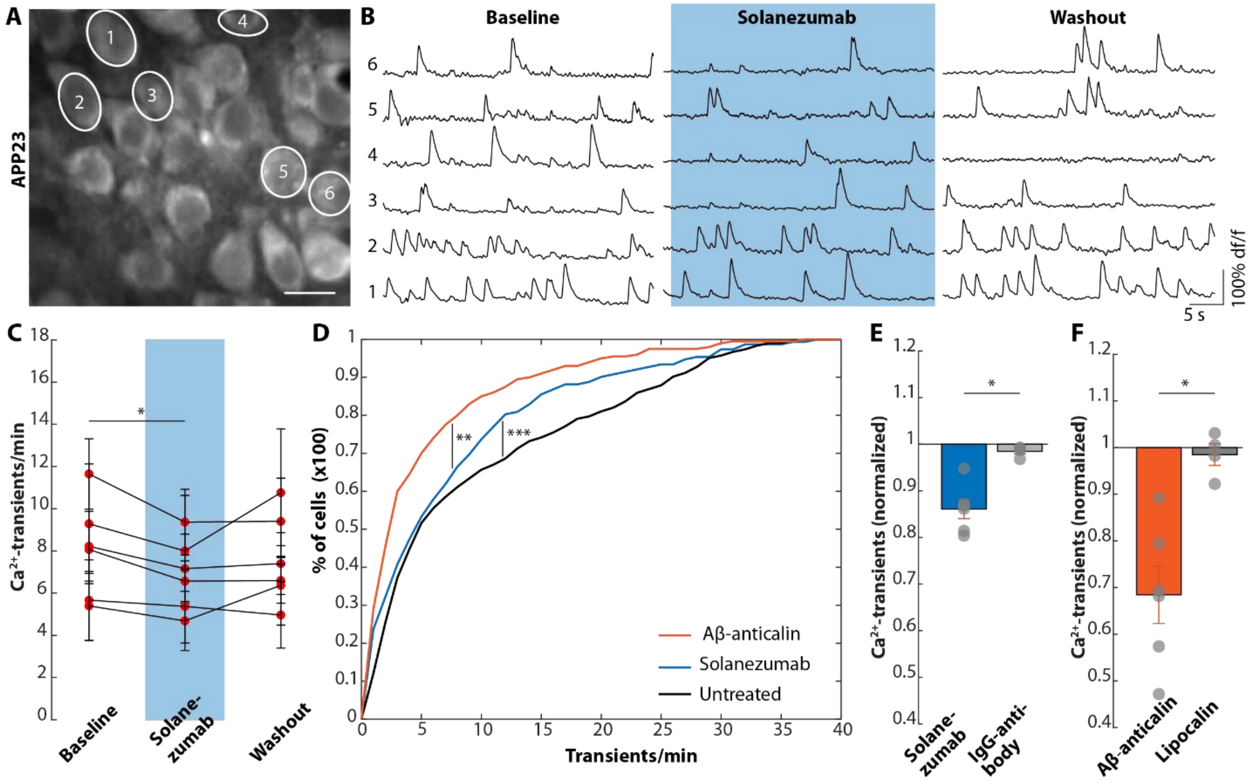

Fig S6.

Solanezumab suppresses neuronal hyperactivity, related to Fig 1: (**A**) Representative two-photon image Ca^{2+}-transients from six hippocampal CA1 pyramidal neurons of a 7-month-old *APP23* mouse under baseline conditions, during the application of Solanezumab (1 µM) and after washout. (**C**) Summary data of the experiment in (B). Red dots represent the mean of one animal (N=6), error bars depict SEM. (**D**) Cumulative probability of the neuronal activity in untreated *APP23* mice (*black*) during Solanezumab (*blue*) or Aβ-anticalin (1µM, *orange*) application. (**E**) Comparison of the average number of Ca^{2+}-transients during the application of Solanezumab (left, N=6 animals) or control IgG in *APP23* mice (*right*, N=4), normalized to the respective baseline activity. (**F**) Same as (E) for the application of Aβ-anticalin (*left*, N=6) or the lipocalin 2 protein (right, N=4). Source data for Fig. S6E and F are provided as a Source Data file * P<0.05, ** P<0.005, ***, P<0.001 Wilcoxon signed rank test (C), Kolmogorow-Smirnow-Test (D), Wilcoxon rank sum test (E and F).

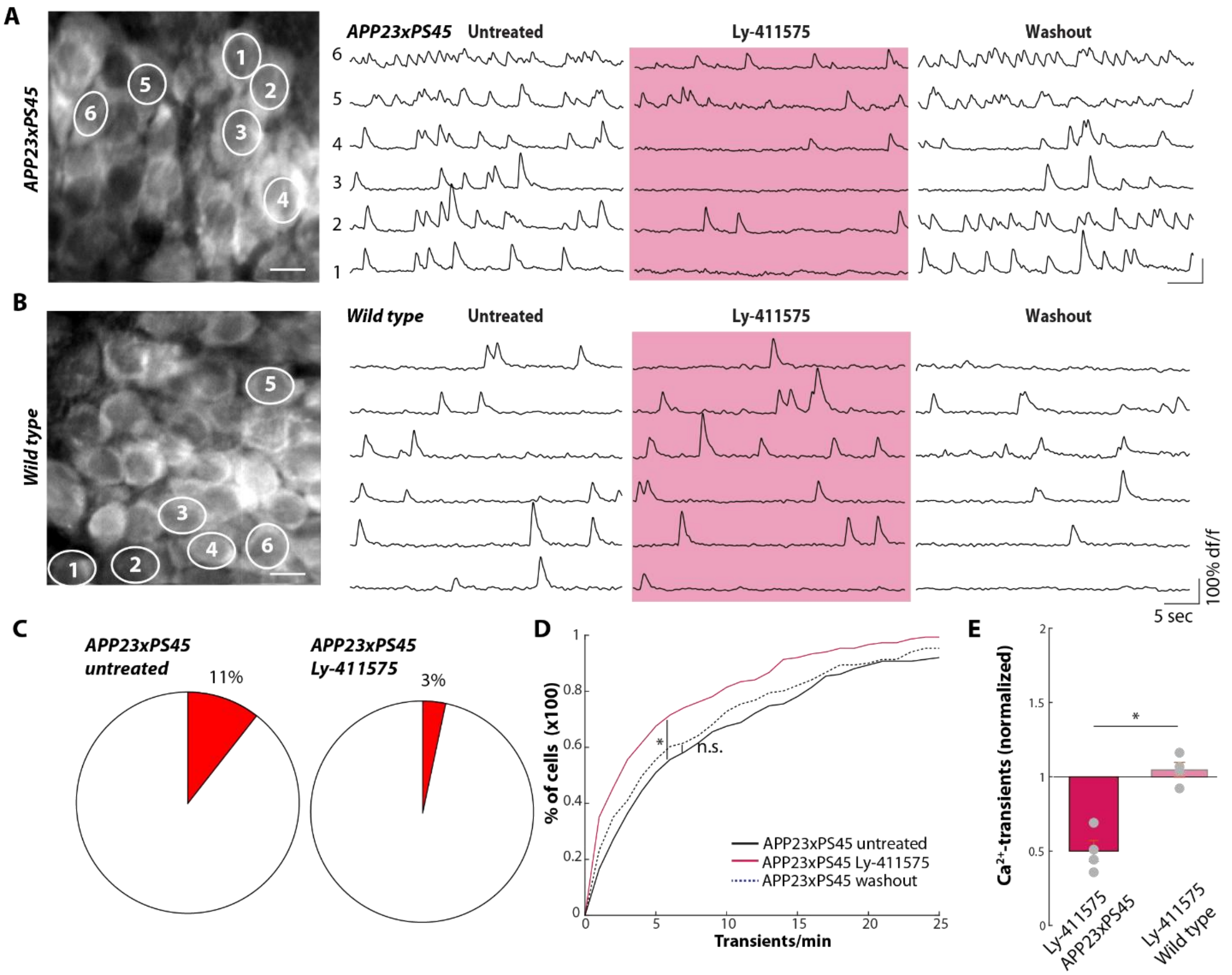

Fig. S7.

Application of the γ-secretase inhibitor Ly-411575 suppresses neuronal hyperactivity, related to Fig 1: (**A**) Representative two-photon image (*left*) and Ca^{2+}-transients from six representative hippocampal CA1 pyramidal neurons of a young *APP23xPS45* mouse under baseline conditions, during the application of Ly-411575 (100 µM) and after washout. (**B**) Same as (A) for a wild-type mouse. (**C**) Pie charts depicting the percentage of hyperactive cells in untreated (*left*) and Ly-411575-treated (*right*) young *APP23xPS45* mice. (**D**) Cumulative probability of the neuronal activity in untreated *APP23xPS45* mice (*black solid*) during Ly-411575-application (*pink*) and after washout (*black dashed*). (**E**) Comparison of the average number of Ca^{2+}-transients during the application of Ly-411575 in young *APP23xPS45* (*left*, N=4 animals) or wild type mice (*right*, N=4), normalized to the respective baseline activity. Source data for Fig. S7E are provided as a Source Data file * $P<0.05$, n.s. not significant, Kolmogorow-Smirnow-Test (D), Wilcoxon rank sum test (E).

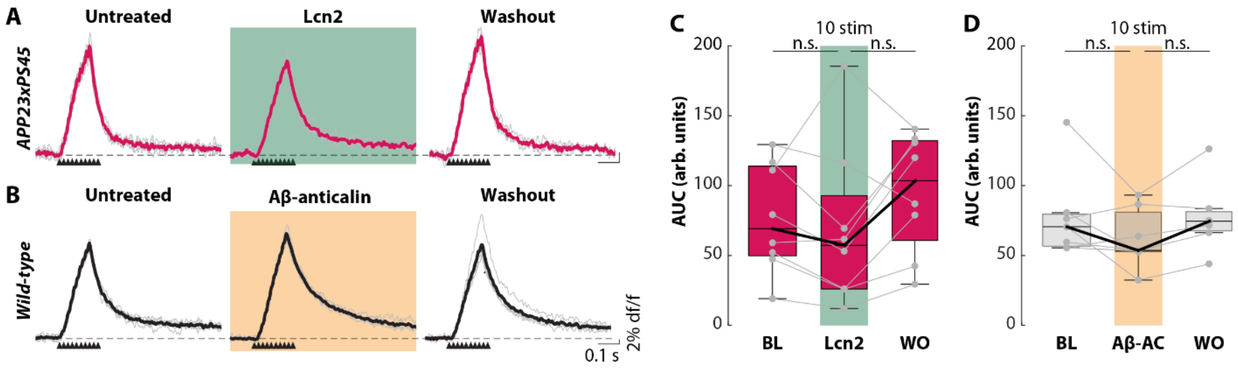

Fig S8.

Wild type lipocalin in *APP23xPS45* or Aβ-anticalin in wild type mice does not affect synaptically-evoked glutamate transients, related to Fig. 2. (**A**) Individual glutamate transients (*grey*) and average evoked by ten stimuli (*pink*) from a young *APP23xPS45* mouse under baseline conditions, during the application of wild type lipocalin (Lcn2, 10 µM) and under washout conditions. (**B**) same as (A) for a slice from a wild type mouse and application of Aβ-anticalin (Aβ-AC, 10 µM). (**C**) Summary data of the experiments in (A) from N=8 slices. Each dot represents the mean AUC of 5 transients from one slice under baseline conditions (BL), during the application of wild type lipocalin and under washout conditions (WO). (**D**) same as (C) for the application of Aβ-anticalin in slices from wild type mice (N=7). N.s. not significant, Wilcoxon signed rank test.

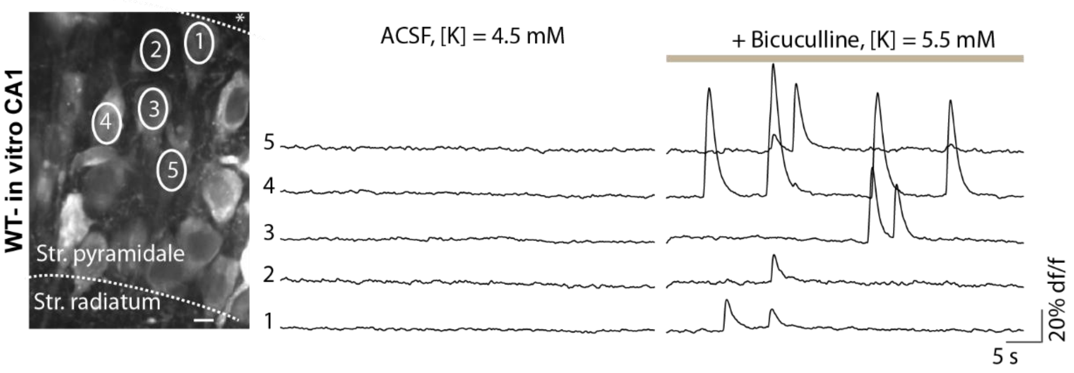

Fig. S9.

Generation of in vivo-like ongoing activity in hippocampal slices, related to Fig 4: *left:* representative two-photon image of the hippocampal CA1 region of a wild type mouse in an acute slice. * marks the edge of stratum oriens, scale bar 5 µm. *Middle:* Ca^{2+}-transients from the five representative neurons cycled in the left panel under baseline conditions, K, potassium. *Right*: Ca^{2+}-transients from the same cells after adding bicuculline (100 µM) in ACSF and increasing the extracellular K^+ concentration to 5.5 mM.

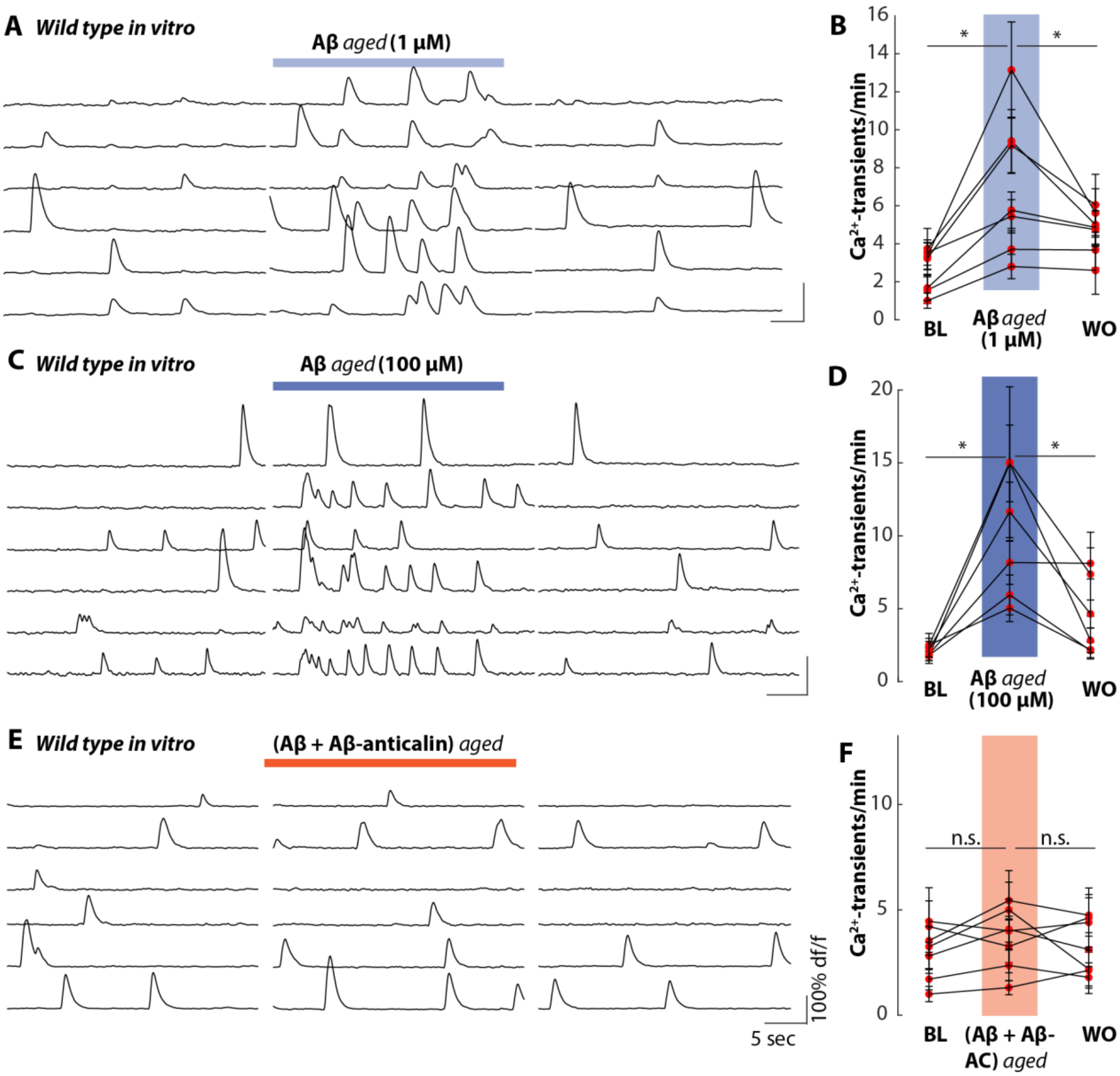

Fig. S10.

In vitro aggregated Aβ monomers induce neuronal hyperactivation in vitro*,* related to Fig. 5: (**A**) Representative Ca^{2+}-traces from six hippocampal CA1 neurons in an acute bicuculline-treated slice from a wild type mouse under baseline conditions (*left*), during the application of putative Aβ(1-40) oligomers (1 µM monomers, incubated for ~90, *middle*) and after a 5 min washout period (*right*). (**B**) Summary data of the *in vitro* experiments in (A). Red dots represent the mean of one animal (N=7 slices), error bars depict SEM. (**C**) same as (A) for the application of 100µM monomers, incubated for 90 min. (**D**) same as (B) for the experiment in (C). (**E**) Same as (A) for the application of 1µM Aβ monomers, incubated for ~90 min in the presence of 1 µM Aβ-anticalin. (**F**) same as (B) for the experiment in (E) *p<0.05, n.s. not significant. Wilcoxon signed-rank test.

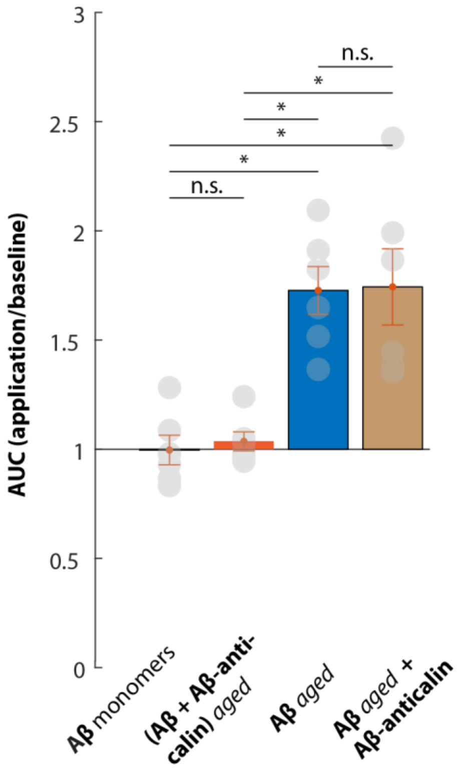

Fig. S11.

AUC analysis of the *in vivo* application experiments in WT mice, related to Fig. 5:

Area under the Ca^{2+}-transients for the application of Aβ monomers (*far left*), Aβ incubated in the presence of the Aβ-anticalin (*left*), aged Aβ (*right*), and aged Aβ after co-incubation with the Aβ-anticalin (*far right*), normalized to the respective baseline. The grey dots represent the mean from an individual mouse (N=6 in each experiment). Source data are provided as a Source Data file. *p<0.05, n.s. not significant. Kruskal-Wallis-Test with Dunn-Sidac post hoc comparison

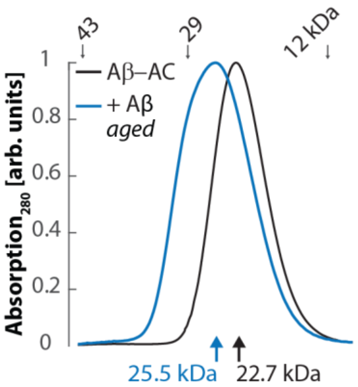

Fig. S12.

The Aβ-anticalin does not dissolve preexisting Aβ-fibrils, related to Fig 5. SEC of Aβ-anticalin alone (*black*) and of 'aged' Aβ incubated for 30 min with the Aβ-anticalin (*blue*). The shift of the peak from 22.7 to 25.5 kDa and the broadening of the peak suggest that the Aβ-anticalin is, to some extent, bound to Aβ monomers but not to larger Aβ aggregates.

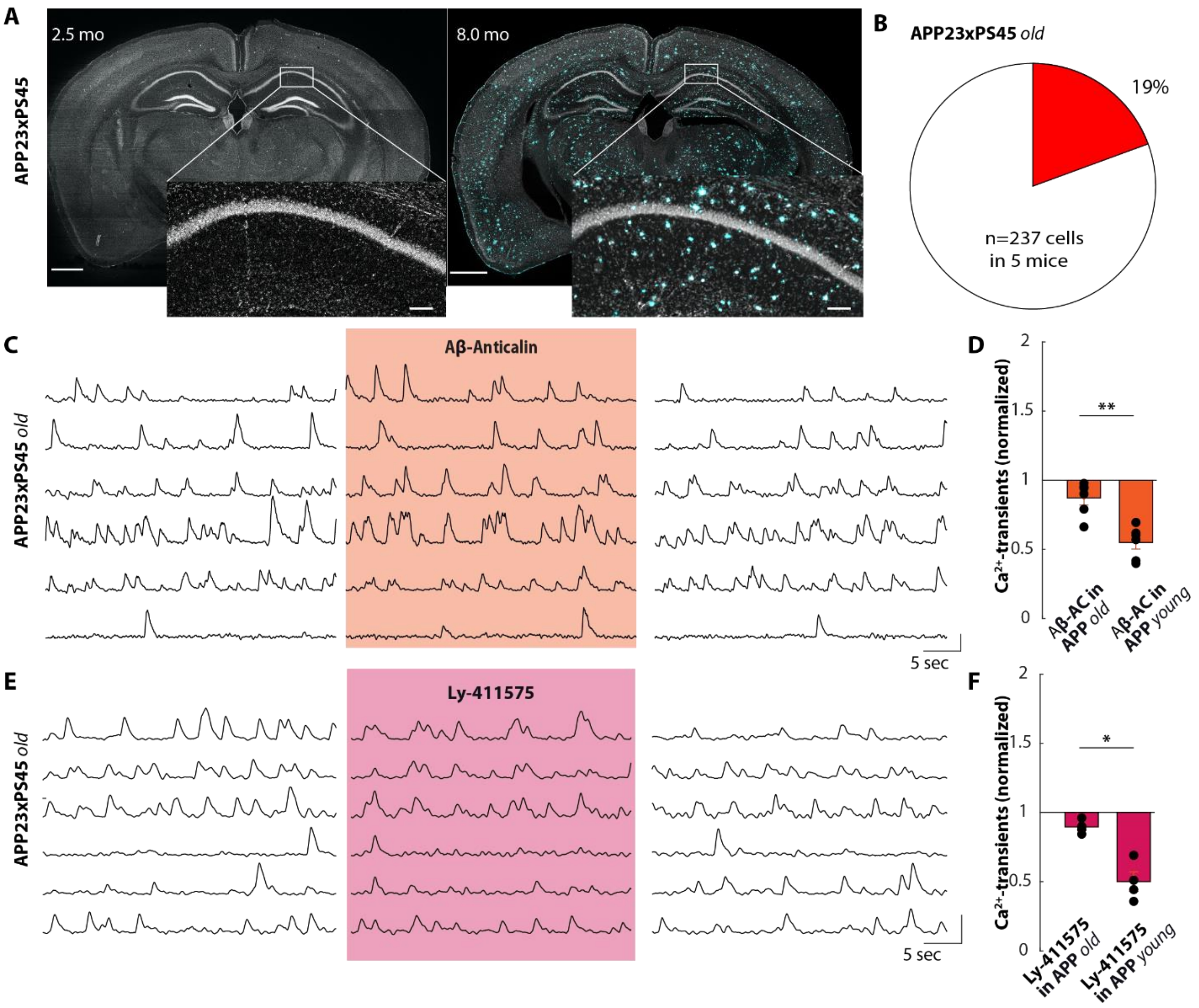

Fig. S13.

No effect of the Aβ-anticalin and Ly-411575 in aged *APP23xPS45* mice, related to Fig. 5:
(**A**) Representative confocal images of coronal brain slices from a young (*left*) and an old (*right*) *APP23xPS45* mouse. Methoxy-X04 staining (*cyan*) and NeuroTrace counterstaining (*grey*). The insets depict the hippocampal CA1 region. Scale bars: 1000µm, inset scale bars: 100µm. (**B**) Pie chart depicting the percentage of hyperactive cells in old *APP23xPS45* mice. (**C**) Ca^{2+}-transients from six representative hippocampal CA1 pyramidal neurons of an old *APP23xPS45* mouse under baseline conditions, during the application of the Aβ-anticalin (10 µM) and after washout. (**D**) Comparison of the average number of Ca^{2+}-transients during the application of the Aβ-anticalin in old (*left*, N=6 animals) or young *APP23xPS45* mice (*right*, N=6), normalized to the respective baseline activity. (**E**) Same as (C) for the application of Ly-411575 (100µM). (**F**) Same as (D) for the application of Ly-411575 in old (*left*, N=4) or young (*right*, N=4) *APP23xPS45* mice. Source data for Fig. S13D and F are provided as a Source Data file **p<0.005*p<0.05, n.s. not significant, Wilcoxon rank sum test (D and F).
